# Respiration Rate Estimation Based on Independent Component Analysis of Accelerometer Data: Pilot Single-Arm Intervention Study

**DOI:** 10.2196/17803

**Published:** 2020-08-10

**Authors:** JeeEun Lee, Sun K Yoo

**Affiliations:** 1 Graduate Program of Biomedical Engineering Yonsei University Seoul Republic of Korea; 2 Department of Medical Engineering Yonsei University College of Medicine Seoul Republic of Korea

**Keywords:** respiration rate, accelerometer, smartphone, independent component analysis, quefrency, mobile phone

## Abstract

**Background:**

As the mobile environment has developed recently, there have been studies on continuous respiration monitoring. However, it is not easy for general users to access the sensors typically used to measure respiration. There is also random noise caused by various environmental variables when respiration is measured using noncontact methods in a mobile environment.

**Objective:**

In this study, we aimed to estimate the respiration rate using an accelerometer sensor in a smartphone.

**Methods:**

First, data were acquired from an accelerometer sensor by a smartphone, which can easily be accessed by the general public. Second, an independent component was extracted to calibrate the three-axis accelerometer. Lastly, the respiration rate was estimated using quefrency selection reflecting the harmonic component because respiration has regular patterns.

**Results:**

From April 2018, we enrolled 30 male participants. When the independent component and quefrency selection were used to estimate the respiration rate, the correlation with respiration acquired from a chest belt was 0.7. The statistical results of the Wilcoxon signed-rank test were used to determine whether the differences in the respiration counts acquired from the chest belt and from the accelerometer sensor were significant. The *P* value of the difference in the respiration counts acquired from the two sensors was .27, which was not significant. This indicates that the number of respiration counts measured using the accelerometer sensor was not different from that measured using the chest belt. The Bland-Altman results indicated that the mean difference was 0.43, with less than one breath per minute, and that the respiration rate was at the 95% limits of agreement.

**Conclusions:**

There was no relevant difference in the respiration rate measured using a chest belt and that measured using an accelerometer sensor. The accelerometer sensor approach could solve the problems related to the inconvenience of chest belt attachment and the settings. It could be used to detect sleep apnea through constant respiration rate estimation in an internet-of-things environment.

## Introduction

### Background

Owing to the rapid recent development of mobile medical monitoring, an increasing number of people desire to manage their health through health care services in daily life. To provide information for health management, it is important to monitor biosignals constantly [1,2]. Among biosignals, respiration is the easiest to measure and provides various types of information about health management based on a number of respiration parameters and respiration patterns [2]. Respiration data collected in daily life can be used as a health care index involving factors such as feedback for sleep apnea syndrome and other issues [3]. In addition, patients could discover abnormalities early through constant monitoring outside of medical facilities and thereby prevent dangerous escalation of their issues [4].

Currently, the sensors used to measure respiration are for medical and experimental use, so they are not easily accessible for general users and their prices are high [5]. In addition, they are of the contact type, requiring users to attach multiple sensors to the body, which makes them difficult to adopt and difficult to use for constant measurement of respiration [6]. To solve these problems, there have been studies on methods to measure respiration using devices such as accelerometers, radar sensors, and thermal cameras [6]. For use with such noncontact monitoring equipment, smartphones allow easy accessibility for general users and integrate easily with mobile monitoring environments [7]. For these reasons, in this study, the number of respirations was calculated using data from an accelerometer sensor passed to a smartphone, which was suitable for monitoring the respiration rate in daily life.

The accelerometer sensor has three axes and has sensitivity related to the degree of inclination and to the direction in which it is resting [8]. Therefore, axial correction is required to use the values from the accelerometer sensor. The typical method for axial correction is to calculate the root sum square of the magnitude [9]. Other methods include the use of the mean along the z-axis, the magnitude of each axis, the calculation of correlation, and the calculation of the average of peak frequency [10]. However, these methods have limitations for removing the uncertainty inherent with accelerometer sensors when they are used in an indoor environment.

There are several methods to estimate respiration. In particular, respiration has been analyzed in the frequency domain using general fast Fourier transform (FFT) and short-time Fourier transform (STFT) [11]. Since respiration is quasi-periodic [12], the dominant component could be found using FFT and STFT. However, when noise is caused from situations, such as power lines and movement, it changes the dominant frequency component [13]. Additionally, after filtration to divide the frequency domain, the number of respirations has been calculated using correlation analysis of filtered respiration signal peak number counters, a Wiener filter, and autocorrelation [11,14-16]. The Wiener filter estimates the signal through assumption of the noise spectrum. When sensors are attached to the body of a subject, the Wiener filter is frequently used to remove body movement [14]. This preprocessing method is difficult to use for measuring respiration accurately owing to its sensitivity to noises. In particular, when respiration is measured with the noncontact method in a mobile environment, various environmental noises occur. Autocorrelation separates the signal and noise. The correlation of a signal can be estimated using a lagged dependent variable [15]. However, it has limitations because respiration is exactly periodic. To solve the problem, we used quefrency selection. A respiration signal is a harmonic component because of its quasi-periodic characteristic. Additionally, a search range related to respiration is set to minimize any noise component.

### Objectives

An accelerometer sensor in a smartphone was used because it is one of the representative internet-of-things (IoT) devices. To reduce the uncertainty caused by external noise, the vector by which the accelerometer sensor independence is maximized was calculated using independent component analysis (ICA). In addition, respiration has regular patterns, so the respiration rate was estimated using quefrency selection reflecting harmonic information. The respiration rate with the suggested method and the respiration rate with the gold standard using a respiration belt were compared to evaluate significance. When the accelerometer sensor in a smartphone is used as an IoT environment, data can be acquired at various locations. Additionally, the specification of the accelerometer sensor is different according to the smartphone device. For this problem, a case study was performed to determine the difference according to location and smartphone. The aim of the study was to estimate the respiration rate based on ICA of an accelerometer sensor.

## Methods

### Data Acquisition

Before data acquisition, recruitment notices were posted on a notice board. Only if a subject agreed to the research, the subject joined the study. Additionally, subjects who were likely to participate in this study were not excluded based on social and economic conditions. The study did not register vulnerable subjects. Before recruiting the subjects, the subjects provided information about their health status, medications, and diseases. Subjects who did not have mental or physical diseases joined the experiment.

This is a single-arm intervention study without a control group and without randomization. Data acquisition was performed in a controlled environment by the laboratory. Each subject signed a written consent form prior to the experiment. The respiration signal was collected while the subject reclined on a bed.

**Figure 1:**
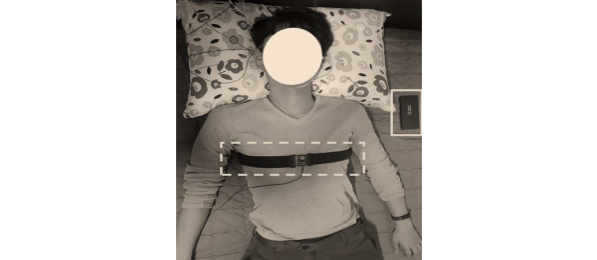
Experimental environment. Solid-line box: Samsung Galaxy S8 accelerometer sensor; dashed-line box: chest belt.

The device used to measure respiration was the accelerometer sensor of the Samsung Galaxy S8 smartphone, and nonlinear sampling was performed at an average of 500 Hz. Respiration signals from a chest belt were measured at the same time as actual respiration signals for comparison with the respiration signals calculated using the accelerometer sensor. Respiration measured from the chest belt was sampled at 500 Hz using BIOPAC MP 150TM equipment. As shown in Figure 1, the accelerometer sensor was located near the left shoulder of the subject, and the chest belt was secured across the chest.

### ICA-Based Accelerometer Calibration

**Figure 2:**
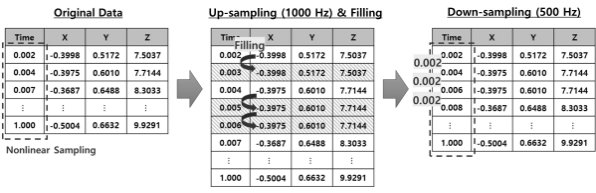
Processing for fixed sampling.

The accelerometer sensor of Samsung Galaxy S8 has a nonlinear sampling rate. To convert this to fixed sampling, time-stamp and accelerometer sensor values were stored simultaneously. Figure 2 shows the process for conversion of the rate of sampling by the accelerometer sensor. The stored original data were up-sampled at 1000 Hz, and missing values were filled in using the same values measured at the previous time stamp. The filled-in missing data were down-sampled again at 500 Hz, which is the same sampling rate used with the original data.

The data measured using the accelerometer sensor contained not only respiration data but also data of various signals, such as motion and external noises. Among these, respiration appeared in the frequency spectrum at less than 0.4 Hz. Thus, respiration signals were preprocessed through a 0.4-Hz low-pass filter [17].

**Figure 3:**
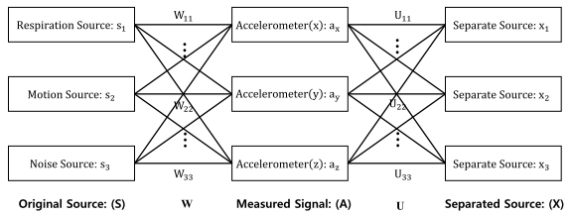
Independent component analysis model for respiration separation.

When an accelerometer sensor is used in an IoT environment, a variety of noises exist in the environment. In addition to respiration, these various noise sources in the environment were also measured (and mixed into the data) by the accelerometer sensor. Therefore, the respiration signals were separated using ICA. Because the original ICA signals were created by other physical processes, it was assumed that they have independent and irregular distributions [18]. Figure 3 presents the ICA model used to distinguish respiration and the signals of the various sources being measured by the accelerometer. It shows the process of separating the sources by estimating *U*.

The separation of sources was calculated using equations 1-3. Equation 1 shows the measured signal (*A*). This signal (*A*) shows the vector measured from the three-axis accelerometer sensor according to time (*t*) and has the value (3 × [*sr* × t]), where *sr* is the sampling rate. In this study, the sampling rate was 500 Hz. To separate the original signals of the accelerometer sensor, equations 2 and 3 were used. In this case, *U* = *W*^−1^ is the mixing matrix, *S* is the original source, and *X* = *S* is the estimated separated source [18].


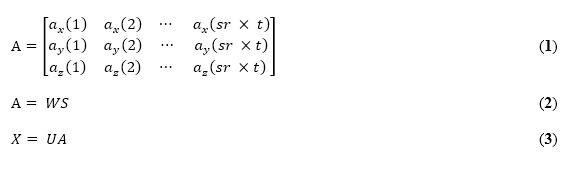


### Quefrency Selection for Respiration Rate Estimation

Respiration is a regular signal, so it is possible to estimate the respiration rate through the estimation of the interval measured by the accelerometer sensor.

For detection of the regularity of *X*, a cepstrum was used. The cepstrum was calculated through inverse Fourier transform. In this way, the harmonic component of signals could be acquired [19,20].

Equation 4 was used for the quefrency selection, which is the harmonic component of *X*, and the term *CA*_max__*_peak_* is the point where the maximum peak of the cepstrum of *X* appears [21]. It is needed to set the search range related to respiration. The respiration-related search range defines searching point (SP). To spot the maximum peak dependent on the respiration signal SP, SP was designated using equation 5. The term *RR*_sec_ shows the respiration rate per second, and SP was designated as (1251-6000) using equation 5.


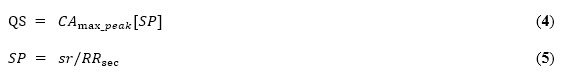


In addition, whether the second harmonic exists is detected through the harmonic component adjacent to the search point. The respiration rate was estimated according to the existence of the second harmonic, and *RR*_min_, the number of respirations per minute, was calculated using equation 6 [21].


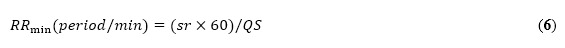


**Figure 4:**
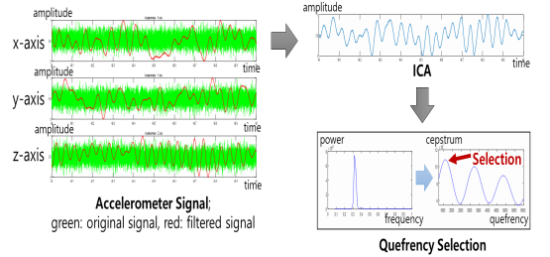
Diagram for respiration rate estimation. ICA: independent component analysis.

Figure 4 shows a diagram displaying how to estimate the respiration rate. The filtered signals from the accelerometer sensor were separated based on ICA. The independent component analyzed using cepstrum and selected the maximum point at the range related to respiration.

## Results

### Data Overview

The study was approved by the Institutional Review Board of Yonsei University health system (IRB number: 4-2018-0411). From April 2018, we enrolled 30 male participants. Among the subjects, the mean age was 26.67 (SD 2.41) years, mean height was 173.8 (SD 5.33) cm, and mean weight was 74.43 (SD 9.52) kg.

### Comparison With the Accelerometer Calibration Method

For analysis of the method presented in this study, the signals acquired from the chest belt were used as the standard. The chest belt signals were segmented with 1-minute epochs, and the number of respirations per minute was calculated by counting the number of maximum peaks. This study presents an accelerometer calibration method using ICA to extract the original signals from the accelerometer sensor.

For assessment of the method presented here, the Pearson correlation (Pearson *r*) was used. With Pearson *r*, the correlation of the number and size of respirations calculated from the actual respiration counts and ICA calibration was determined. Because *r* is close to −1 or 1, it is very similar to the real respiration rate.

The ICA methods proposed for accelerometer calibration of each axis, the root sum square (RSS) of each axis, and principal component analysis (PCA) methods (which were used previously) were verified using Pearson *r*. Each axis is affected by tilting and direction [8]. Since the signal is acquired while the subject and smartphone are set on the bed, it is important to confirm which axis is more sensitive after preprocessing such as filtering. If one axis is correlated with respiration, the axis has to be selected to reduce calculation. The RSS is calculated using the square root sum of each axis, and it is frequently used for calibration of the accelerometer sensor [9]. PCA is similar to ICA as one of the dimension reduction methods. It finds new principal axes while preserving the variance and transforms data from a high-dimensional space to a low-dimensional space without linear correlation [22].

**Figure 5:**
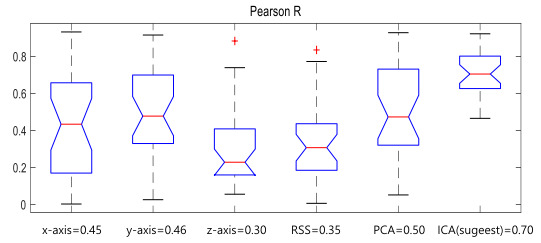
Skeletal box-and-whisker plot according to the calibration method. ICA: independent component analysis; PCA: principal component analysis; RSS: root sum square.

Figure 5 shows Pearson *r* skeletal box-and-whisker plots for respiration rate estimation according to each calibration method. In the skeletal box-and-whisker plots, the red line is the median, and the skeletal box is represented from the first to third quartiles of Pearson *r*. The minimum and maximum of Pearson *r* without outliers are shown as whiskers with end caps [23]. When calibrated with ICA, it has the highest correlation (0.7). In addition, ICA has a lower difference in the range between the maximum and minimum values compared with other values, so the result with it is more stable than those with other calibration methods.

In this study, statistical evaluation was performed on whether the ICA calibration presented with the use of the Pearson *r* value was significant compared with other methods (x-axis, y-axis, z-axis, RSS, and PCA). Prior to statistical evaluation, visual regularity verification was performed using a quantile-quantile plot. When identified using a quantile-quantile plot, all the data were hard to consider as linear, so a nonparametric test was performed. Because significance was evaluated according to each method using the same subjects, a Wilcoxon signed-rank test was used. Table 1 presents the statistical results of the Wilcoxon signed-rank test. The Pearson *r* of the respiration rate estimation according to the ICA method was significant (*P*<.001) compared with the other methods.

**Table 1:** Statistical evaluation of the calibration method using the Wilcoxon rank-sum test.

Method	W	z	*P* value
x-axis	647	−3.95	<.001
y-axis	635	−4.13	<.001
z-axis	533	−5.64	<.001
RSS^a^	567	−5.14	<.001
PCA^b^	713	−2.98	.003

^a^RSS: root sum square.

^b^PCA: principal component analysis.

### Comparison With Conventional Algorithms

After the accelerometer sensor was calibrated using ICA, conventional methods to extract the respiration rate estimation and the proposed quefrency selection method were verified using Pearson *r*. The compared conventional methods were processed with filtering of the acquisition data. There were three methods as follows: peak count, spectral peak transition, and autocorrelation. In the three compared methods, the peak count determines the peaks from the ICA calibration data. To estimate the respiration dominant frequency, the highest spectral peak transition is selected using FFT. The selected spectral peak transition shows the signal period. Autocorrelation is calculated using correlation with the lagged values [15].

**Figure 6:**
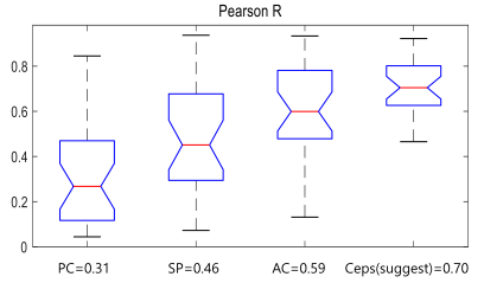
Skeletal box-and-whisker plot according to the conventional algorithm. AC: autocorrelation; PC: peak count; SP: spectral peak transition.

Figure 6 shows Pearson *r* skeletal box-and-whisker plots for the respiration rate estimation according to each conventional method. When the respiration rate was estimated by quefrency selection, it was determined to have the highest correlation value (0.7). In addition, there was little difference in the range between the maximum and minimum values compared with other methods, so it was more stable than other conventional algorithms.

In this study, statistical evaluation was performed to determine whether the quefrency selection presented with use of the Pearson *r* value was significantly different from the other methods (peak count, spectral peak transition, and autocorrelation). The statistical results of the Wilcoxon signed-rank test in Table 2 show that the respiration rate estimation by the quefrency selection method has more relevant results.

**Table 2:** Statistical evaluation of conventional algorithms using the Wilcoxon rank-sum test.

Method	W	z	*P* value
Peak count	521	−5.82	<.001
Spectral peak transition	680	−3.47	<.001
Autocorrelation	774	−2.08	.04

### Comparison With the Chest Belt

Table 3 shows the results from comparing the average respiration count per minute acquired using the chest belt (*R.R*_belt_) with that acquired using the accelerometer sensor (*R.R*_est_) for each subject. The difference in the respiration rate per minute between the two sensors was at the most two times the respiration count. When the peaks were counted using the chest belt, a peak counting error occurred with the respiratory waveforms of a half cycle at the first and the last respiration. On the other hand, the quefrency selection was independent in respiratory waveforms of a half cycle, so errors were reduced in the detection of the respiration rate. Table 3 shows that there was no significant difference in the performance of respiration rate estimation between the accelerometer sensor and the chest belt (W=842, z=−1.11, *P*=.27).

**Table 3:** Results of respiration rate estimation.

Number	*R.R*_belt_^a^	*R.R*_est_^b^	Difference
1	15	17	2
2	18	18	0
3	16	16	0
4	16	17	1
5	17	17	0
6	18	18	0
7	16	15	1
8	18	18	0
9	17	19	2
10	18	20	2
11	17	18	1
12	15	16	1
13	17	18	1
14	16	17	1
15	18	20	2
16	16	16	0
17	15	15	0
18	15	17	2
19	16	16	0
20	16	17	1
21	17	16	1
22	16	15	1
23	17	16	1
24	16	16	0
25	15	16	1
26	16	17	1
27	15	15	0
28	18	18	0
29	17	16	1
30	17	17	0

^a^*R.R*_belt_: average respiration count per minute acquired using the chest belt.

^b^*R.R*_est_: average respiration count per minute acquired using the accelerometer sensor.

In addition, the statistical results of the Wilcoxon signed-rank test were used to determine whether the differences in the respiration count acquired from the chest belt and from the accelerometer sensor were significant. The *P* value of the difference in the respiration count acquired from the two sensors was .27, which was not significant. This indicates that the number of respiration counts measured using the accelerometer sensor was not different from that measured using the chest belt. Therefore, it is possible to use an accelerometer sensor for estimation of the respiration rate, instead of a chest belt.

**Figure 7:**
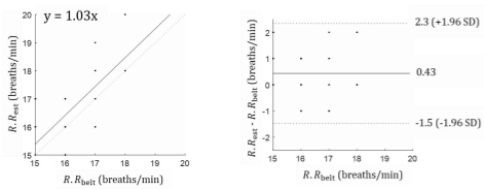
Bland-Altman results between the respiration belt and accelerometer sensor. *R.R*_est_: average respiration counts per minute acquired using the accelerometer sensor; *R.R*_belt_: average respiration counts per minute acquired using the chest belt.

A Bland-Altman analysis was performed to evaluate the performance of respiration rate estimation from the accelerometer sensor compared with the respiration belt. The Bland-Altman analysis showed a correlation between the accelerometer sensor and respiration belt (Figure 7A). Figure 7B shows the Bland-Altman results for the mean difference and the bias of the 95% confidence interval [24]. In Figure 7, the correlation coefficient was 0.7. The Bland-Altman results indicate that the mean difference was 0.43, with less than one breath per minute, and that the respiration rate was at the 95% limits of agreement. The accelerometer sensor could produce results ranging from a 2.3 breaths per minute overestimation to 1.5 breaths per minute underestimation.

### Case Study

Here, *Loc*_up_ is the case in which the smartphone was located next to the left shoulder of the subject and *Loc*_down_ is the case in which it was located under the left foot. Table 4 shows the results of respiration estimation according to location. The difference in respiration count per minute by location was at minimum zero times and at maximum one time. In most cases, no difference in the respiration count occurred, which indicates that the quefrency selection method using an accelerometer sensor has low sensitivity to location (W=97, z=−0.65, *P*=.52).

**Table 4:** Results of respiration rate estimation by location.

Number	*Loc*_up_^a^	*Loc*_down_^b^	Difference
1	17	17	0
2	18	19	1
3	15	15	0
4	16	17	1
5	16	15	1
6	16	16	0
7	18	18	0
8	17	17	0
9	18	18	0
10	17	17	0

^a^*Loc*_up_: smartphone is located next to the left shoulder.

^b^*Loc*_down_: smartphone is located under the left foot.

**Figure 8:**
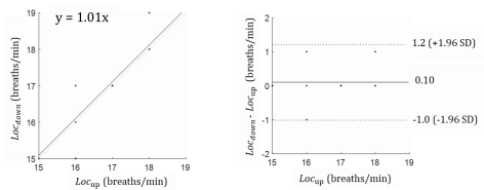
Bland-Altman results according to sensor location. *Loc*_down_: smartphone is located under the left foot; *Loc*_up_: smartphone is located next to the left shoulder.

In Figure 8, the correlation coefficient is 0.93. The Bland-Altman results indicated that the mean difference was 0.1, with less than one breath per minute, and that the respiration rate was at the 95% limits of agreement. The accelerometer sensor could produce results ranging from a 1.2 breaths per minute overestimation to 1.0 breath per minute underestimation.

There is a difference in the accelerometer sensor of different types of smartphones. Therefore, to identify the difference according to the type of smartphone, a case study was performed in 10 subjects. The smartphones used for the case study were Samsung Galaxy S8 (smartphone 1) and Samsung Galaxy S7 (smartphone 2). Table 5 shows the results of respiration rate estimation using each smartphone. The difference in the respiration count per minute for each smartphone type was at minimum zero times and at maximum one time, which indicates that there was no relevant difference in the results with these two smartphones.

The Wilcoxon signed-rank test was used to determine statistically whether the estimated respiration count was significantly different according to the type of smartphone and location. The *P* value of the difference in the estimated respiration count by location was .52, which was not significant. This shows that there was no significant difference in the respiration count estimated at different locations. The *P* value of the difference in the estimated respiration count by smartphone type was .88, which was also not significant (W=103, z=−0.16). This shows that there was no significant difference in the respiration count estimated using the two different smartphones.

**Table 5:** Results of respiration rate estimation by smartphone.

Number	Smartphone 1^a^	Smartphone 2^b^	Difference
1	18	18	0
2	17	18	1
3	18	18	0
4	18	19	1
5	17	17	0
6	17	17	0
7	18	18	0
8	18	18	0
9	18	18	0
10	19	19	0

^a^Smartphone 1: Samsung Galaxy S8.

^b^Smartphone 2: Samsung Galaxy S7.

**Figure 9:**
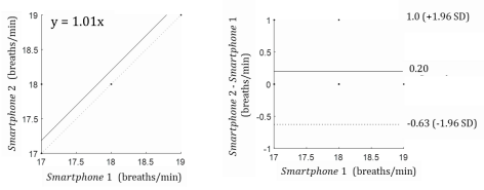
Bland-Altman results according to smartphone type. Smartphone 1: Samsung Galaxy S8; smartphone 2: Samsung Galaxy S7.

In Figure 9, the correlation coefficient is 0.79. The Bland-Altman results indicate that the mean difference was 0.2, with less than one breath per minute, and that the respiration rate was at the 95% limits of agreement. The accelerometer sensor could produce results ranging from a 1.0 breath per minute overestimation to 0.63 breaths per minute underestimation.

## Discussion

### Principal Findings

In this study, respiration counts were estimated based on data from an accelerometer sensor in a smartphone. During recent advances in the mobile medical monitoring environment, a variety of smart devices have been developed [2,3]. There is also an increasing desire to manage health by measuring and analyzing data using such smart devices [1,5]. In particular, the development of various wearable devices, such as smartphones and smart bands, provides the potential to acquire various types of health care information such as movement and heartbeat. Respiration is a biosignal directly related to body activity [3,5]. The estimation of respiration can prevent dangerous incidents by predicting diseases and detecting sleep apnea [4]. However, the number of smart devices able to present respiration rates is limited, and the sensors that can accurately detect respiration are not easy to use in daily life. Thus, the purpose of this study was to estimate respiration counts using an accelerometer sensor in a smartphone that is easy to access by normal people.

This study acquired signals using the accelerometer sensor in a smartphone for a long time to identify the feasibility of constantly estimating respiration during natural motion. Because the accelerometer sensor in a smartphone samples nonlinearly, linear sampling was imposed. The accelerometer sensor is a three-axis type, which requires calibration. In this study, components that maximize independence from the signals acquired from the three axes of the accelerometer sensor were distinguished using ICA, and signals showing the range of respiration were extracted. When the statistical significance of the difference in results for the conventional method and Pearson *r* of the respiration counts estimated from the chest belt and accelerometer sensor were determined, ICA showed a significant result.

Lastly, accelerometer sensors are greatly affected by environmental noises. However, while such noises have random characteristics, respiration has regularity, and this regularity has harmonic components. Thus, respiration rates were estimated using quefrency selection. It was confirmed that there was a relevant difference in the data acquired using Pearson *r* for the conventional method and quefrency selection, as well as statistical verification. It was also confirmed that the performance of respiration rate estimation was excellent when quefrency selection was applied to signals acquired from the accelerometer sensor. This was determined through verification of the difference and statistical significance of the number of respiration counts calculated using the chest belt and the proposed (accelerometer) method.

The use of the accelerometer sensor in a smartphone is a noncontact method and has the advantage of constant respiration monitoring. This enables easy measurement of respiration in daily life. However, when it is used in an actual environment, a number of environmental variables exist. Specifically, the same user could put the smartphone at different locations during the measurements, and the performances of embedded sensors could also be different according to smartphone type. Therefore, case studies were performed according to sensor location and smartphone type. The results indicated that respiration rate detection is possible independent of location and smartphone type.

### Limitations

The situation in which respiration rate estimation is needed the most is during sleep [3,4]. Diseases can be predicted and emergency situations can be judged by detecting sleep apnea. Therefore, in this study, respiration was estimated while the subjects were lying down. The feasibility for long-term estimation was also confirmed. In the future, signals during sleep could be acquired and analyzed to apply actual respiration rate estimation during sleep. However, the experiment environment was controlled. During the respiration rate estimation, subjects could change position and lay laterally or in the prone position. Therefore, a further study about position change is needed for application of the approach in a real environment.

### Conclusions

In this study, respiration rates were estimated using data from the accelerometer sensor of a smartphone as an IoT device. This study showed differentiation of the respiration rate estimation achieved through ICA calibration and quefrency selection. The respiration estimation sensors that are currently used are not easy to access and not easy to use in daily life owing to the need for multiple sensors with direct contact. However, smartphones are easy to use in daily life and some are equipped with accelerometer sensors, which makes them suitable for respiration rate estimation. There is a difference in performance according to the calibration method used with the accelerometer sensor. The accuracy of the respiration rate estimation can be enhanced when independent components are detected from three-axis ICA signals and quefrency selection is applied. This new approach could solve the problems related to the inconvenience of electrode attachment and equipment settings that affect respiration rate estimation. Furthermore, it could be used to detect sleep apnea through constant respiration rate estimation in an IoT environment.

## Abbreviations

FFT: fast Fourier transform
ICA: independent component analysis
IoT: internet-of-things
PCA: principal component analysis
RSS: root sum square
STFT: short-time Fourier transform

## References

[1] Kristiani DG, Triwiyanto T, Nugraha PC, Irianto BG, Titisari D, Syaifudin (2019). The Measuring of Vital Signs Using Internet Of Things Technology (Heart Rate And Respiration).

[2] Faust-Christmann CA, Taetz B, Zolynski G, Zimmermann T, Bleser G (2019). A Biofeedback App to Instruct Abdominal Breathing (Breathing-Mentor): Pilot Experiment. JMIR Mhealth Uhealth.

[3] Schade MM, Bauer CE, Murray BR, Gahan L, Doheny EP, Kilroy H, Zaffaroni A, Montgomery-Downs HE (2019). Sleep Validity of a Non-Contact Bedside Movement and Respiration-Sensing Device. J Clin Sleep Med.

[4] Nam Y, Kim Y, Lee J (2016). Sleep Monitoring Based on a Tri-Axial Accelerometer and a Pressure Sensor. Sensors (Basel).

[5] Bari R, Adams RJ, Rahman MM, Parsons MB, Buder EH, Kumar S (2018). rConverse: Moment by Moment Conversation Detection Using a Mobile Respiration Sensor. Proc ACM Interact Mob Wearable Ubiquitous Technol.

[6] Al-Khalidi FQ, Saatchi R, Burke D, Elphick H, Tan S (2011). Respiration rate monitoring methods: a review. Pediatr Pulmonol.

[7] Bhattacharya R, Bandyopadhyay N, Kalaivani S (2017). Real time Android app based respiration rate monitor.

[8] Wannenburg J, Malekian R (2017). Physical Activity Recognition From Smartphone Accelerometer Data for User Context Awareness Sensing. IEEE Trans. Syst. Man Cybern, Syst.

[9] Wang F, Chan H, Hsu M, Lin C, Chao P, Chang Y (2018). Threshold-based fall detection using a hybrid of tri-axial accelerometer and gyroscope. Physiol Meas.

[10] Liu G, Guo Y, Zhu Q, Huang B, Wang L (2011). Estimation of respiration rate from three-dimensional acceleration data based on body sensor network. Telemed J E Health.

[11] Novak P, Novak V (1993). Time/frequency mapping of the heart rate, blood pressure and respiratory signals. Med. Biol. Eng. Comput.

[12] Sharma H, Sharma K (2018). ECG-derived respiration using Hermite expansion. Biomedical Signal Processing and Control.

[13] Omlin X, Crivelli F, Heinicke L, Zaunseder S, Achermann P, Riener R (2016). Effect of Rocking Movements on Respiration. PLoS One.

[14] Fedotov AA, Akulov SA, Akulova AS (2017). Motion artifacts reduction in wearable respiratory monitoring device.

[15] Kwasniewska A, Ruminski J, Szankin M (2019). Improving Accuracy of Contactless Respiratory RateEstimation by Enhancing Thermal Sequences withDeep Neural Networks. Applied Sciences.

[16] Huang R (2018). Contact-free breathing rate monitoring with smartphones: a sonar phase approach. Auburn University.

[17] Norton MP, Nelson FC (1990). Fundamentals of Noise and Vibration Analysis for Engineers. The Journal of the Acoustical Society of America.

[18] Kim BS, Yoo SK (2006). Motion Artifact Reduction in Photoplethysmography Using Independent Component Analysis. IEEE Trans. Biomed. Eng.

[19] Heo H, Sung D, Lee K (2013). Note onset detection based on harmonic cepstrum regularity.

[20] Skowronski MD, Shrivastav R, Hunter EJ (2015). Cepstral Peak Sensitivity: A Theoretic Analysis and Comparison of Several Implementations. J Voice.

[21] Lee J, Yoo SK (2020). Radar-Based Detection of Respiration Rate with Adaptive Harmonic Quefrency Selection. Sensors (Basel).

[22] Bates A, Ling MJ, Mann J, Arvind DK (2010). Respiratory rate and flow waveform estimation from tri-axial accelerometer data.

[23] Spitzer M, Wildenhain J, Rappsilber J, Tyers M (2014). BoxPlotR: a web tool for generation of box plots. Nat Methods.

[24] Martin Bland J, Altman D (1986). Statistical Methods For Assessing Agreement Between Two Methods of Clinical Measurement. The Lancet.

